# Supplementary material for: Bio-inspired design of ice-retardant devices based on benthic marine invertebrates: the effect of surface texture
Source: PeerJ. 2014 Sep 23;2:e588. doi: 10.7717/peerj.588 (PMC4179385; doi:10.7717/peerj.588)
Supplement: Supplemental Information 3 — This provides the data and R code used to generate Figs. 3–5. It also provides plots showing dots for all the raw measurements in response to a request from Reviewer 2 (P Cziko). [file peerj4sem.pdf › peerj4/peerj4sem.pdf]

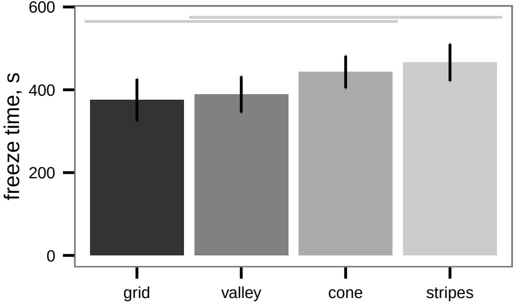

600
400
200
0
freeze time, s
grid
valley
cone
stripes
