# Supplementary material for: Bio-inspired design of ice-retardant devices based on benthic marine invertebrates: the effect of surface texture
Source: PeerJ. 2014 Sep 23;2:e588. doi: 10.7717/peerj.588 (PMC4179385; doi:10.7717/peerj.588)
Supplement: Supplemental Information 3 — This provides the data and R code used to generate Figs. 3–5. It also provides plots showing dots for all the raw measurements in response to a request from Reviewer 2 (P Cziko). [file peerj5asem.pdf › peerj5/peerj5asem.pdf]

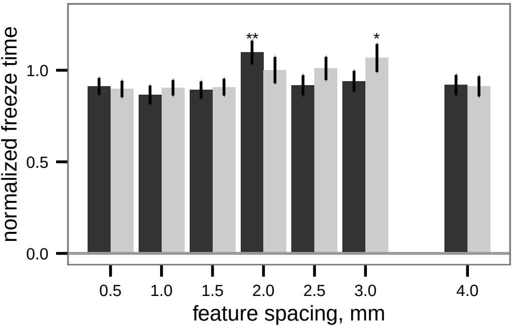

normalized freeze time
0.0
0.5
1.0
**
*
0.5
1.0
1.5
2.0
2.5
3.0
4.0
feature spacing, mm
