# Supplementary material for: Bio-inspired design of ice-retardant devices based on benthic marine invertebrates: the effect of surface texture
Source: PeerJ. 2014 Sep 23;2:e588. doi: 10.7717/peerj.588 (PMC4179385; doi:10.7717/peerj.588)
Supplement: Supplemental Information 3 — This provides the data and R code used to generate Figs. 3–5. It also provides plots showing dots for all the raw measurements in response to a request from Reviewer 2 (P Cziko). [file peerj5braw.pdf › peerj5/peerj5braw.pdf]

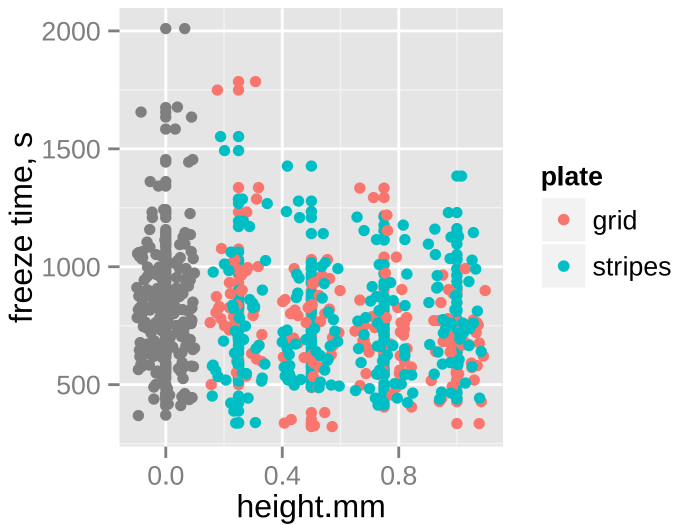

2000
1500
1000
500
freeze time, s
0.0
0.4
0.8
height.mm
plate
grid
stripes
