# Supplementary material for: Bio-inspired design of ice-retardant devices based on benthic marine invertebrates: the effect of surface texture
Source: PeerJ. 2014 Sep 23;2:e588. doi: 10.7717/peerj.588 (PMC4179385; doi:10.7717/peerj.588)
Supplement: Supplemental Information 3 — This provides the data and R code used to generate Figs. 3–5. It also provides plots showing dots for all the raw measurements in response to a request from Reviewer 2 (P Cziko). [file peerj5bsem.pdf › peerj5/peerj5bsem.pdf]

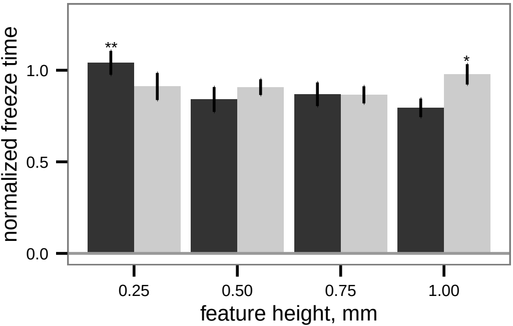
normalized freeze time
1.0
0.5
0.0
**
*
0.25
0.50
0.75
1.00
feature height, mm
