# Supplementary material for: Bio-inspired design of ice-retardant devices based on benthic marine invertebrates: the effect of surface texture
Source: PeerJ. 2014 Sep 23;2:e588. doi: 10.7717/peerj.588 (PMC4179385; doi:10.7717/peerj.588)
Supplement: Supplemental Information 3 — This provides the data and R code used to generate Figs. 3–5. It also provides plots showing dots for all the raw measurements in response to a request from Reviewer 2 (P Cziko). [file peerj3sem.pdf › peerj3/peerj3sem.pdf]

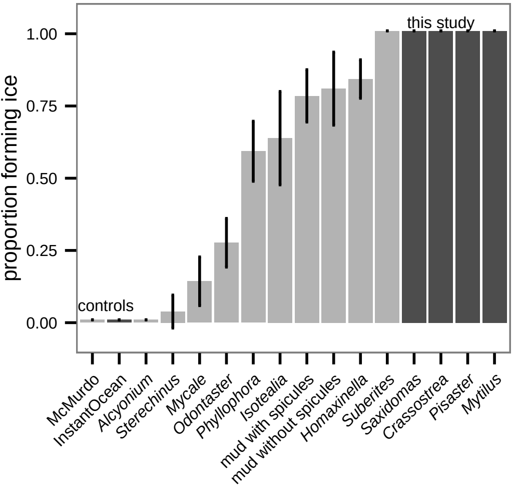

proportion forming ice
1.00
0.75
0.50
0.25
0.00
controls
this study
McMurdo
InstantOcean
Alcyonium
Sterechinus
Mycale
Odontaster
Phyllophora
Isotealia
mud with spicules
mud without spicules
Homaxinella
Suberites
Saxidomas
Crassostrea
Pisaster
Mytilus
